# Nitrogen fertilizer modulated the effect of drought priming on photosynthesis, antioxidant defense, nitrogen metabolism, yield in summer maize

**DOI:** 10.3389/fpls.2026.1792261

**Published:** 2026-04-13

**Authors:** Jinhui Xie, Chen Ru, Yang Liu, Longzhe Quan, Chuanliu Xie, Xiaotao Hu

**Affiliations:** 1 School of Mechanical and Vehicle Engineering, Anhui Agricultural University, Hefei, China; 2 Key Laboratory of Agricultural Soil and Water Engineering in Arid and Semiarid Areas, Ministry of 7 Education, Northwest A&F University, Yangling, China

**Keywords:** antioxidant, drought priming, N metabolism, nitrogen, root morphology

## Abstract

**Introduction:**

Post-anthesis drought stress is a major constraint on the growth and yield formation of summer maize. Although drought priming can alleviate drought stress damage, whether increased nitrogen (N) application can enhance this beneficial regulation remains unclear.

**Methods:**

This study aimed to explore the regulatory mechanisms underlying the combined effects of drought priming and N fertilizer on photosynthetic characteristics, antioxidant systems, N metabolism, root morphology, and yield formation in summer maize.

**Results and discussion:**

Compared with the single drought treatment (N1D), the combined treatment of drought priming and moderate N application (N2P) significantly increased the maximum photochemical efficiency of PSII (F_v_/F_m_), actual photochemical quantum yield of PSII (ΦPSII), and net photosynthetic rate (P_n_) by 13.8%, 38.1%, and 32.4%, respectively, and effectively improved the chlorophyll, flavonoid, and anthocyanin indices. N fertilizer enhanced the priming-induced activation of the antioxidant system, with the N2P treatment increasing superoxide dismutase (SOD), peroxidase (POD), and catalase (CAT) activities by 28.9%-57.1%. Under the regulation of priming and N fertilizer, the expression of N metabolism-related genes remained at relatively high levels, leading to significantly elevated nitrate reductase (NR) activity and aboveground N accumulation. Meanwhile, root length density, root surface area density, and root dry weight density increased by 31.6%, 22.5%, and 13.9%, respectively. These coordinated improvements in physiology and morphology optimized the yield components, thus resulting in a 16.2% grain yield advantage for N2P treatment over N1D, while achieving the highest drought tolerance. Collectively, drought priming combined with N2 application effectively improved maize yield by synergistically improving N metabolism, protecting the photosynthetic system, enhancing antioxidant capacity, and promoting root architecture development. This provides an agronomic strategy to obtain stable, high-yield, and efficient resource use in summer maize in arid areas.

## 1 Introduction

Against the backdrop of global climate change, the rising frequency and intensity of extreme weather events, such as high temperatures and droughts, have severely threatened agricultural production (Li et al., 2025a). As a crucial grain crop in China, the stable production of summer maize is directly linked to national food security. However, the key growth stages of summer maize often coincide with seasonal droughts, making drought stress the primary abiotic constraint limiting high and stable yields (Bheemanahalli et al., 2022; Sheoran et al., 2022). Drought stress impairs multiple physiological functions including plant water balance, inhibits photosynthesis, and interferes with metabolic homeostasis, ultimately leading to significant yield losses (Bheemanahalli et al., 2022).

Drought stress impacts summer maize throughout its growth cycle, disrupting developmental processes, interfering with physiological metabolism, and ultimately reducing yield formation. Water deficit impairs leaf water status, directly inhibiting light energy absorption and carbon assimilation efficiency, thereby reducing the photosynthetic rate (Chen et al., 2023). To maintain cellular homeostasis, plants activate antioxidant defense systems and osmotic adjustment mechanisms to scavenge excess reactive oxygen species (ROS) (Ru et al., 2023; Gelaw and Sanan-Mishra, 2024). However, prolonged stress exceeding the regulatory threshold aggravates membrane lipid peroxidation and damages cell membrane integrity (Gelaw and Sanan-Mishra, 2024). Additionally, drought stress suppresses the activity of key enzymes responsible for nitrogen uptake and assimilation, hindering nitrogen translocation to photosynthetic organs and grains. This ultimately manifests as stunted growth, reduced biomass accumulation, diminished sink capacity, and impaired grain-filling efficiency during the reproductive stage, leading to substantial yield losses (Dong et al., 2022; Yue et al., 2022). Therefore, developing robust agronomic strategies to improve drought tolerance is of paramount importance for safeguarding summer maize production.

Drought priming can induce physiological adaptation and stress memory in crops, thereby conferring enhanced tolerance to subsequent drought events (Wang et al., 2023). Research has demonstrated that priming optimizes stomatal movement, reducing water transpiration while maintaining CO_2_ supply, slowing the degradation of photosynthetic pigments, and facilitating the retention of high photosynthetic capacity (Tankari et al., 2021). Furthermore, drought priming can induce the production osmolytes (e.g., proline and soluble sugars) and activate antioxidant enzyme systems, including superoxide dismutase and peroxidase, enabling plants to initiate defense mechanisms more rapidly when encountering drought and mitigating membrane lipid peroxidation damage (Abdallah et al., 2017; Ru et al., 2022). Drought priming also helps stabilize the activity of key nitrogen assimilation enzymes, alleviating drought-induced inhibition of nitrogen uptake and translocation, thereby providing sustained nitrogen support for plant growth (He et al., 2024). However, the effectiveness of drought priming is influenced by factors such as crop variety, stress intensity, and soil fertility. Under conditions of insufficient nitrogen supply, the stress resistance response triggered by priming may be constrained by limited nitrogen resources (Ullah et al., 2022).

Nitrogen (N) is an essential macronutrient for crop growth and development and is directly involved in photosynthetic carbon assimilation and the transport and distribution of substances (Fathi, 2022). Under drought stress conditions, N fertilizer exerts a significant positive regulatory effect on improving crop stress resistance. As the material basis for osmotic adjustment substances and antioxidant enzyme synthesis. N can effectively enhance cellular osmotic adjustment capacity and ROS scavenging efficiency, thereby maintaining the structural integrity of membrane systems (Iqbal et al., 2020; Ru et al., 2023). Under drought stress, rational N application improves maize root growth and increases the number of deep roots and lateral root branching, thereby enhancing the water uptake capacity (Li et al., 2025b). Furthermore, N can optimize the transport and distribution patterns of photosynthates, promote the targeted accumulation of assimilates in the ears, and ultimately increase the number of kernels per ear and 1000-kernel weight (Biswas and Ma, 2016; Noor et al., 2023). However, the interaction between N availability and drought tolerance remains unclear. Excessive N application may increase crop sensitivity to drought, particularly under severe water stress (Lin et al., 2012). High N application can restrict root extension into deeper soil layers, leading to exacerbated leaf wilting and reduced N-use efficiency (Li et al., 2025). Therefore, the interaction between drought stress and N fertilizer and their regulatory mechanisms require further investigation.

Previous research on drought tolerance has predominantly focused on single regulatory measures. The combination of drought priming and N management is expected to significantly alleviate yield losses through the synergistic effect of stress signal induction and N supply. This study hypothesizes that the synergistic application of elevated N and drought priming could enhance drought tolerance and yield in summer by improving physiological traits. The objectives of this research were: (1) to elucidate the regulatory effects of the interaction between drought priming and N application on photosynthesis, antioxidant system, N metabolism, and yield of summer maize; and (2) to evaluate the synergistic enhancement mechanism underlying their interaction on drought tolerance, yield, and water-N use efficiency.

## 2 Materials and methods

### 2.1 Experimental materials

**Figure 1:**
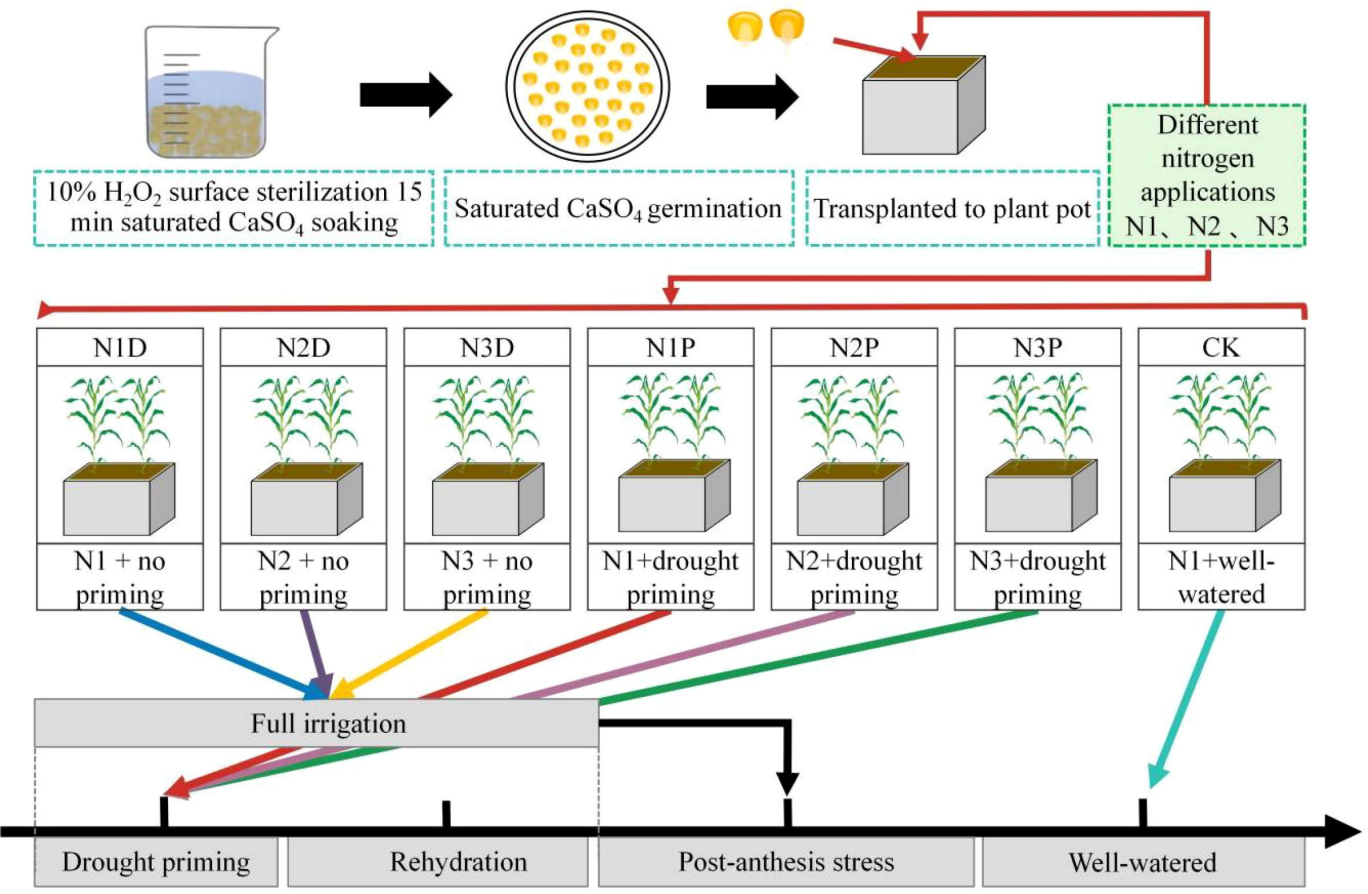
Experimental design and flowchart. N1D, N1 + no priming + post-anthesis stress; N2D, N2 + no priming + post-anthesis stress; N3D, N3 + no priming + post-anthesis stress; N1P, N1 + drought priming + post-anthesis stress; N2P, N2 + drought priming + post-anthesis stress; N3P, N3 + drought priming + post anthesis stress; CK, N1 + no priming + well-watered.

The experiment was conducted in 2022 at the Water-Saving Irrigation Experimental Station of the Key Laboratory of Agricultural Soil and Water Engineering in Arid Areas, Ministry of Education, Northwest A&F University. The widely cultivated summer maize hybrid Zhengdan 958 (Zheng 58 × Chang 7-2), developed by the Henan Academy of Agricultural Sciences, was used in this study. It is characterized by compact plant architecture, strong lodging resistance, efficient nutrient and water use, good stress tolerance, and high, stable yield. Following surface sterilization in a 10% hydrogen peroxide solution for 15 min, the seeds were rinsed repeatedly with distilled water to ensure complete removal of the disinfectant. To promote germination, the treated seeds were soaked in saturated CaSO^4^ solution for 6 h, placed on sterile gauze, and germinated in an incubator at 25 °C. Seedlings with a root length of 1–2 cm were selected for uniformity and transplanted into pots (55 cm × 40 cm × 60 cm, length × width × height) with two seedlings per pot. The pots were filled with air-dried topsoil (0–30 cm layer) from the experimental site, which was sieved through a 0.5 mm mesh. The main physicochemical properties of the soil were as follows: field capacity (FC) of 25.78%, pH of 7.7, organic matter content of 12.71 g·kg^-1^, nitrate nitrogen of 11.24 mg·kg^-1^, ammonium nitrogen of 6.58 mg·kg^-1^, available phosphorus of 18.93 mg·kg^-1^, and available potassium of 159.60 mg·kg^-1^. Sowing was conducted in early June and harvesting in early October (**Figure 1**).

### 2.2 Experimental design

In this study, three nitrogen (N) application levels were established using urea (containing 46% N) as the nitrogen fertilizer: N1 (20 g N m^-2^), N2 (25 g N m^-2^), and N3 (30 g N m^-2^). Among them, N1, determined with reference to the conventional local N application, was set as the control, while N2 and N3 were designated as increased N application treatments. N fertilizer was split-applied as follows: 40% as basal fertilizer at sowing, 30% at the jointing stage, and 30% at the large trumpet stage. Basal applications of P (P_2_O_5_) and K (K_2_O) were applied at rates of 90 kg·ha^-1^ and 120 kg·ha^-1^, respectively. To ensure uniform seedling establishment, all pots were well irrigated to maintain soil moisture at 75%–85% FC from sowing until the initiation of drought priming. At the V12 stage, plants assigned to the priming treatment were subjected to a 10−day period of moderate drought (50%–60% FC). After the priming period, all plants were rewatered to 75%–85% FC until the anthesis stage. Subsequently, a 15-day post-anthesis drought stress (50%–60% FC) was applied to investigate the interactive effects of drought priming and N supply. After the stress treatment, all plants were rewatered (75%–85% FC) until they reached maturity. Non-primed plants were only exposed to the 15-day post-anthesis drought stress, with adequate irrigation maintained during other growth stages. The control group (CK) received well-watered throughout the entire growth period. Seven treatments were established: N1D (N1 + no priming + post-anthesis drought stress), N2D (N2 + no priming + post-anthesis drought stress), N3D (N3 + no priming + post-anthesis drought stress), N1P (N1 + drought priming + post-anthesis drought stress), N2P (N2 + drought priming + post-anthesis drought stress), N3P (N3 + drought priming + post-anthesis drought stress), and CK (N1 + no priming + well-watered). Each treatment included 15 replicate pots (**Figure 1**).

After emergence, all potted plants were grown in a rain-shelter environment. Throughout the experimental period, the soil moisture content was monitored using a portable soil moisture meter (Takeme-10^®^, Mianyang, China). When the soil moisture content decreased to the lower limit, water was replenished precisely to the target upper limit using a measuring cylinder, and the amount of irrigation was documented.

### 2.3 Plant sampling

The last fully expanded leaves were sampled on the 7th (D7) and 14th (D14) days of post-anthesis drought stress. Chlorophyll fluorescence and membrane stability index (MII) were measured immediately using fresh intact leaves. For the other assays, leaf samples were immediately flash-frozen in liquid nitrogen and stored at −80 °C until further analysis. The frozen samples were used for the determination of hydrogen peroxide (H_2_O_2_), superoxide anion (O_2_^-^), malondialdehyde (MDA), superoxide dismutase (SOD), peroxidase (POD), catalase (CAT), ascorbate peroxidase (APX), soluble sugars (SS), proline (Pro), nitrate reductase (NR) activity, and the expression of key N metabolism genes. All physiological and biochemical parameters were determined with five biological replicates, each replicate being measured from an independent leaf from a different plant.

### 2.4 Parameter measurement and methods

#### 2.4.1 Photosynthetic and chlorophyll fluorescence parameters

At D14, the net photosynthetic rate (P_n_, µmol·m^-2^·s^-1^), stomatal conductance (g_s_, mol·m^-2^·s^-1^), and transpiration rate (T_r_, mmol·m^-2^·s^-1^) of leaves were determined using a portable photosynthesis system (LI-6400^®^, LI-COR, USA). Three leaves were randomly selected per treatment, and three readings were recorded for each leaf. Chlorophyll fluorescence parameters were measured using a Fluorcam^®^ portable chlorophyll fluorescence imager (Handy Fluorcam FC 1000-HC, Photon Systems Instruments, Czech Republic), including the maximum photochemical efficiency of PSII (F_v_/F_m_), the actual photochemical quantum yield of PSII (ΦPSII), and the non-photochemical quenching coefficient (NPQ).

#### 2.4.2 N nutrition-related parameters

The chlorophyll (µg·cm^-2^), anthocyanin, flavonoid, and nitrogen balance indices were assessed using a handheld Dualex^®^ instrument (Model DX16641, Force‑A, France). The nitrogen balance index was derived as the ratio of the chlorophyll to flavonoid indices.

#### 2.4.3 Reactive oxygen species content and membrane stability index

The contents of hydrogen peroxide (H_2_O_2_, mol·g^-1^·FW), superoxide anion (O_2_^–^, µg·g^-1^·FW), and malondialdehyde (MDA, mmol·g^-1^·FW) were determined according to established methods. Briefly, H_2_O_2_ was extracted from 0.3 g leaf tissue with ice-cold 0.1% trichloroacetic acid, and the supernatant was reacted with potassium phosphate buffer and KI before measuring the absorbance at 390 nm (Okuda et al., 1991). The O_2_^–^ content was assayed as described by Elstner and Heupel (1976). MDA content, an indicator of lipid peroxidation, was quantified using the thiobarbituric acid (TBA) reaction method (Du and Bramlage, 1992), with absorbance measured at 532 nm, 600 nm, and 450 nm.

The membrane injury index (MII, %) was determined as described by Deshmukh et al. (1991). Briefly, leaf samples (0.3 g) were sectioned into uniform segments and divided into two aliquots for analysis. Each portion was immersed in 10 mL of deionized water in a separate test tube. One set of tubes was incubated at 30 °C for 30 min, while the other set was heated in a boiling water bath (100 °C) for 20 min. The electrical conductivity of the solution in each tube was measured after the treatments and recorded as E_1_ (30 °C) and E_2_ (100 °C). The MII was calculated as the ratio of E_1_ to E_2_.

#### 2.4.4 Antioxidant enzyme activities

Superoxide dismutase (SOD, Unit·mg^-1^·protein), peroxidase (POD, Unit·mg^-1^·protein), and catalase (CAT, Unit·mg^-1^·protein) activities were measured according to the methods of Beauchamp and Fridovich (1971); Zhang (1992), and Aebi (1984), respectively, with the absorbance determined at 560 nm, 470 nm and 240 nm by a spectrophotometer for the activity assay of each enzyme. Ascorbate peroxidase (APX, Unit·g^-1^·FW) activity was assayed following Nakano and Asada (1981), with activity quantified by spectrophotometrically monitoring ascorbate oxidation at 290 nm.

#### 2.4.5 Soluble sugar and proline content

The proline content (µg·g^-1^·DW) was assayed according to Bates et al. (1973). Briefly, 1 mL of leaf extract was sequentially reacted with 2 mL of acid-ninhydrin reagent and 2 mL of glacial acetic acid. The solution was heated for 1 h in a boiling water bath, rapidly cooled on ice, and then extracted with 3.5 mL of toluene prior to absorbance reading at 520 nm. The soluble sugar content (mg·g^-1^·DW) was analyzed using the anthrone-sulfuric acid method (Yemm and Willis, 1954). Specifically, 100 μL of extract was combined with 3 mL of anthrone reagent, heated at 95 °C for 10 min, cooled to room temperature, incubated for 20 min, and the absorbance was recorded at 625 nm.

#### 2.4.6 Activities and gene expression of N metabolism enzymes

Nitrate reductase (NR, EC 1.6.6.1) activity (µg·g^-1^·DW) was assayed using the protocol established by Wang et al. (2013) with some modifications. The nitrogen content in aboveground organs was measured using a Futura^®^ continuous flow analysis system (Alliance Instruments, France). Aboveground nitrogen accumulation (ANA) was determined by multiplying aboveground nitrogen content by aboveground dry weight. Total RNA was isolated from leaf samples, and quantitative real-time polymerase chain reaction (qRT-PCR) analysis was performed based on the protocol reported by Van Oosten et al. (2017). The molybdenum cofactor biosynthesis gene (GRMZM2G067176) served as the internal control, and the relative expression levels of target genes were computed using the 2^-ΔΔCT^ method. All primers were designed to amplify cDNA fragments of 100–140 bp at an annealing temperature of 58 ± 2 °C, with the detailed primer sequences, Tm values and product lengths listed in **Supplementary Figure S1**. The amplification efficiencies of the different primer sets differed by less than 5%.

#### 2.4.7 Summer maize growth traits

The leaf area per plant (cm^2^·plant^-1^) was determined using a leaf area meter (LI-3100^®^, LI-COR, Lincoln, NE, USA). Plant height (cm) was determined for five selected plants per treatment using a measuring tape. The aboveground biomass (g·plant^-1^) was collected and oven-dried at 75 °C until a constant weight was achieved. At maturity, five plants per treatment were collected. The root samples were gently rinsed with deionized water to remove adhering soil particles. Root samples were scanned using a flatbed scanner, and the total root length and root surface area were determined using a root analysis system (WinRHIZO^®^, Regent Instruments Inc., Canada). After scanning, root samples were dried at 75 °C to a constant weight for the determination of root dry weight. Root length density (RLD, m·m^-3^), root surface area density (RSAD, m^2^·m^-3^), and root dry weight density (RDWD, g·m^-3^) were calculated as the ratios of total root length, total root surface area, and total root dry weight to the total soil volume of the pot, respectively.

#### 2.4.8 Yield and water-nitrogen use efficiency

At maturity, four pots per treatment were sampled for yield component analysis, including ear length (cm), ear diameter (cm), kernel number per ear, and 1000-kernel weight (g). Grain yield per plant (g) was calculated after adjusting the grain moisture content to the standard 14%. Water use efficiency for yield (WUE_y_, g·g^-1^ N), water use efficiency for biomass (WUE_b,_ g·g^-1^ N), nitrogen use efficiency for yield (NUE_y_, g·g^-1^ N), nitrogen use efficiency for biomass (NUE_b_, g·g^-1^ N) and harvest index (HI, %) were calculated using Equations 1–5 as follows:

(1) $${\text{WUE}}_{\text{y}}=\text{Grain yield}/\text{irrigation water}$$

(2) $${\text{WUE}}_{\text{b}}=\text{Biomass accumulation}/\text{irrigation water}$$

(3) $${\text{NUE}}_{\text{y}}=\text{Grain yield}/\text{aboveground nitrogen accumulation}$$

(4) $${\text{NUE}}_{\text{b}}=\text{Biomass accumulation}/\text{aboveground nitrogen accumulation}$$

(5) HI=Grain yield/biomass

### 2.5 Data analysis

Statistical analyses were performed using SPSS 22.0 (version 22.0). Duncan’s multiple range test (*p* < 0.05) was applied to compare treatment means. A two-way ANOVA was conducted to evaluate the main and interactive effects of drought priming and nitrogen application. Pearson correlation analysis was conducted to examine the relationships among the physiological variables. A heatmap was constructed to visualize the differential responses of the parameters across N levels and priming treatments. All figures were generated using Origin 2025 (OriginLab, Northampton, MA, USA).

## 3 Results

### 3.1 Aboveground growth and root morphology

**Figure 2:**
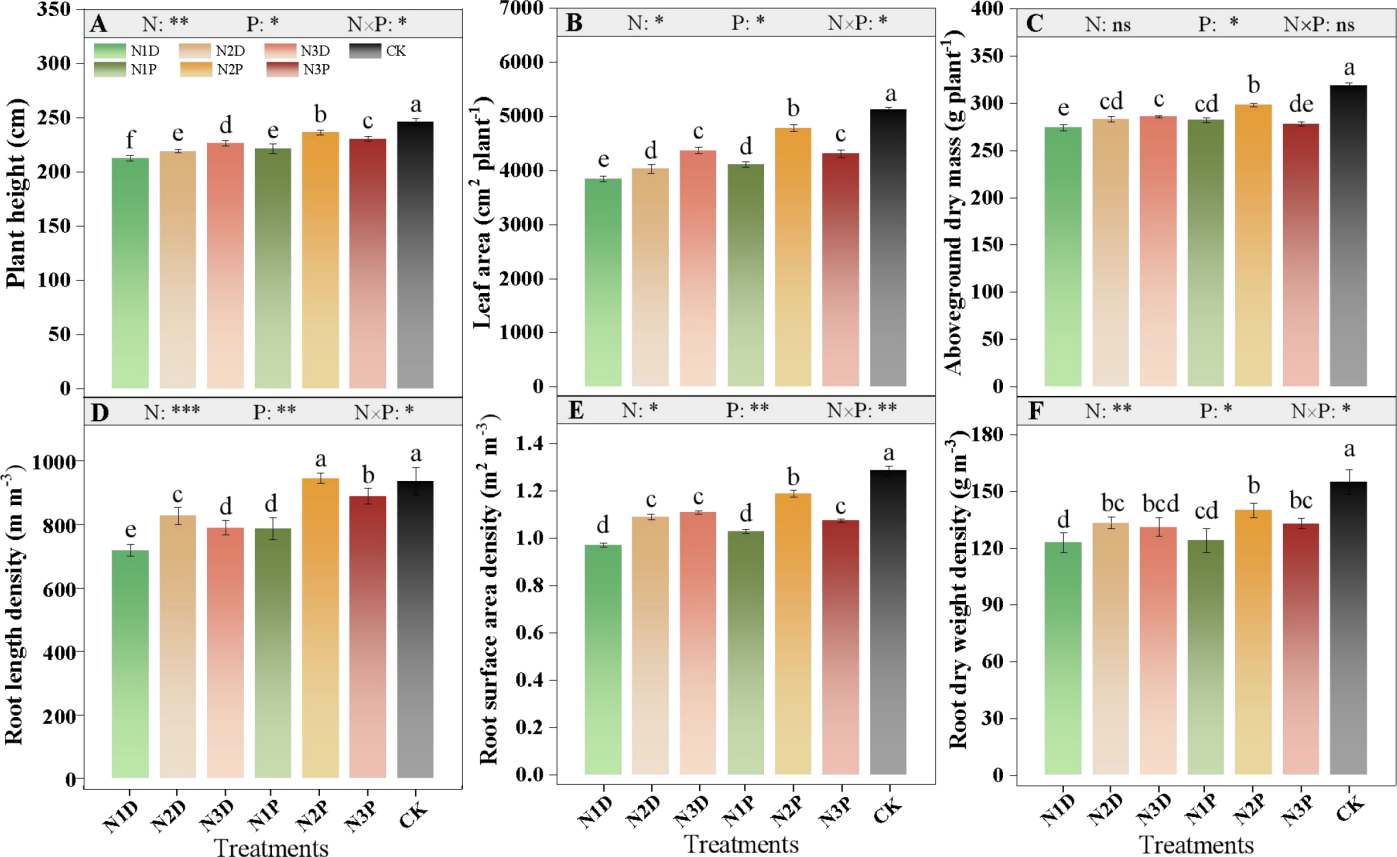
Effects of nitrogen fertilizer and drought priming on plant height **(A)**, leaf area **(B)**, aboveground dry mass ADM, **(C)**, root length density (RLD, **(D)**), root surface area density RSAD, **(E)**, and root dry weight density RDWD, **(F)** in summer maize. N1D, N1 + no priming + post-anthesis stress; N2D, N2 + no priming + post-anthesis stress; N3D, N3 + no priming + post-anthesis stress; N1P, N1 + drought priming + post-anthesis stress; N2P, N2 + drought priming + post-anthesis stress; N3P, N3 + drought priming + post anthesis stress; CK, N1 + no priming + well-watered; N, nitrogen; P, drought priming. Data represent mean ± SD (n = 5). *p < 0.05, **p < 0.01, ***p < 0.001, ns is nonsignificant (*p* > 0.05). Different lowercase letters indicate significant differences (*p* < 0.05).

Plant height, leaf area, and root density were primarily influenced by the nitrogen fertilizer (N) × drought priming (P) interaction (**Figure 2**). Drought stress significantly inhibited the aboveground growth of maize. Compared with the control, plant height, leaf area, and aboveground dry mass (ADM) in the N1D treatment decreased by 13.7%, 24.9%, and 14.0%, respectively. Under no priming conditions, the three aboveground growth traits increased with increasing N application, whereas the N2P treatment maintained significantly higher values than the N3D treatment (**Figures 2A–C**). Under the same N application, primed plants demonstrated a significantly greater root lenght density (RLD) than non-primed plants; the RLD of the N2P and N3P treatments exhibited increases of 31.6% and 23.8%, respectively (**Figure 2D**). For root surface area density (RSAD), no significant difference was detected among the N2D, N3D, and N3P treatments, but all displayed significantly lower values than the N2P treatment. Compared with the control, the RSAD declined by 24.4% under the N1D, whereas the reduction was only 7.5% under the N2P (**Figure 2E**). Except for N1P, a greater root dry weight density (RDWD) was observed in all other treatments compared with the N1D (**Figure 2F**).

### 3.2 Yield and water-nitrogen use efficiency

#### 3.2.1 Yield and its components

**Figure 3:**
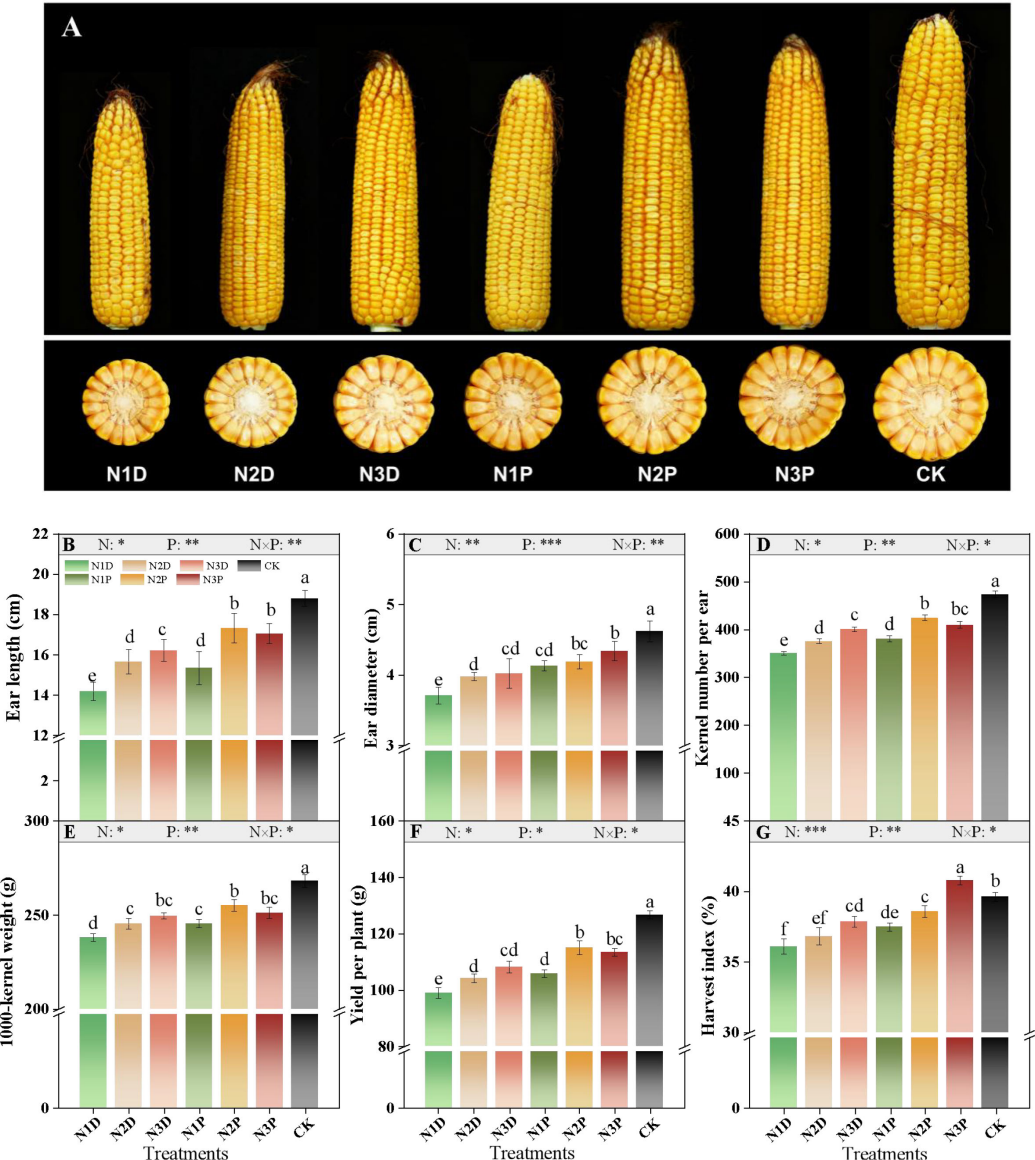
Effects of nitrogen fertilizer and drought priming on maize ear **(A)**, ear length **(B)**, ear diameter **(C)**, kernel number per ear **(D)**, 1000-kernel weight **(E)**, yield per plant **(F)**, and harvest index **(G)** in summer maize. N1D, N1 + no priming + post-anthesis stress; N2D, N2 + no priming + post-anthesis stress; N3D, N3 + no priming + post-anthesis stress; N1P, N1 + drought priming + post-anthesis stress; N2P, N2 + drought priming + post-anthesis stress; N3P, N3 + drought priming + post-anthesis stress; CK, N1 + no priming + well-watered; N, nitrogen; P, drought priming. Data represent mean ± SD (n=4). *p < 0.05, **p < 0.01, ***p < 0.001, ns is nonsignificant (*p* > 0.05). Different lowercase letters indicate significant differences (*p* < 0.05).

Changes in grain yield, yield components, and harvest index (HI) depended on nitrogen fertilizer (N), drought priming (P), and their interaction effects (**Figure 3**). Different treatments had significant impacts on maize ears (**Figure 3A**). Ear diameter and harvest index (HI) increased with increasing N supply, with primed plants exhibiting more pronounced increases than non-primed plants. Ear diameter and HI in the N3P treatment were 1.17- and 1.13-fold greater than those in the N1D treatment, respectively (**Figure 3C, G**). Under no priming conditions, ear length, kernel number per ear, and 1000-kernel weight increased with increasing N application. Under priming conditions, these parameters showed a quadratic response to increasing N supply, with the N2P treatment showing increases of 22.0%, 21.1%, and 7.1%, compared with N1D (**Figures 3B, D**, and **E**). Combined increased N supply and drought priming effectively mitigated yield reduction. The N2P and N3P treatments yielded similar grain yields, surpassing the N1D treatment by 16.2% and 14.6%, respectively (**Figure 3F**).

#### 3.2.2 Water and nitrogen use efficiency of maize

**Figure 4:**
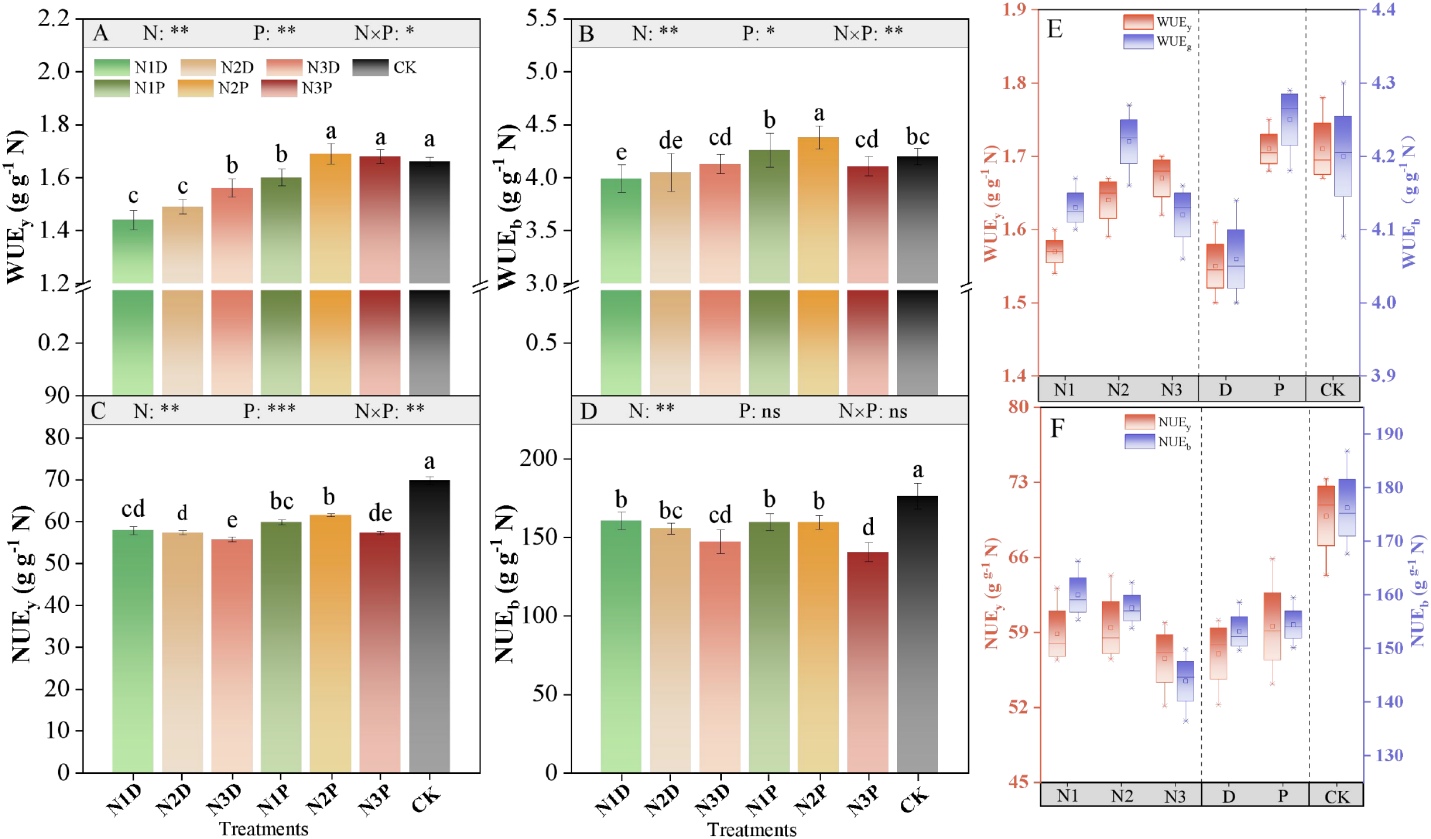
Effects of nitrogen fertilizer and drought priming on water use efficiency for yield WUE_y_, **(A)** water use efficiency for biomass WUE_b_, **(B)** nitrogen use efficiency for yield NUE_y_, **(C)** N use efficiency for biomass NUE_b_, **(D)** in summer maize. Changes in WUE **(E)** and NUE **(F)** under different nitrogen applications and drought priming conditions. N1D, N1 + no priming + post-anthesis stress; N2D, N2 + no priming + post-anthesis stress; N3D, N3 + no priming + post-anthesis stress; N1P, N1 + drought priming + post-anthesis stress; N2P, N2 + drought priming + post-anthesis stress; N3P, N3 + drought priming + post anthesis stress; CK, N1 + no priming + well-watered; N, nitrogen; P, drought priming. Data represent mean ± SD (WUE_y_ and NUE_y_, n = 4; WUE_b_ and NUE_b_, n=5). *p < 0.05, **p < 0.01, ***p < 0.001, ns is nonsignificant (*p* > 0.05). Different lowercase letters indicate significant differences (*p* < 0.05).

The nitrogen fertilizer (N) × drought priming (P) interaction significantly affected water use efficiency for yield (WUE_y_), water use efficiency for biomass (WUE_b_), and nitrogen use efficiency for yield (NUE_y_), whereas nitrogen use efficiency for biomass (NUE_b_) was primarily influenced by N (**Figure 4**). Under drought stress, WUE_y_ and WUE_b_ decreased by 13.3% and 5.0%, respectively, compared with the control. These two indices in the N3D treatment were 8.3% and 3.5% higher than those in the N1D treatment, respectively. Drought priming further amplified the beneficial regulatory effect of N fertilizer, resulting in 17.4% and 16.7% increases in WUE_y_ in the N2P and N3P treatments compared with the N1D, respectively. Appropriately increasing N supply under drought priming improved WUE_b_, with the N2P treatment being significantly higher than that of the control (**Figures 4A, B**). Compared with the control, NUE_y_ and NUE_b_ decreased by 17.1% and 9.0%, respectively, under drought stress. The N2P treatment achieved a 6.3% higher NUE_y_ than the N1D, whereas NUE_b_ did not differ significantly among the N1P, N2P, and N1D treatments (**Figures 4C, D**). WUE_y_ generally increased with rising N application, whereas NUE_y_ and NUE_b_ exhibited an opposite trend. Under drought priming conditions, WUE_y_, WUE_b_, NUE_y_, and NUE_b_ were higher than those under non-primed conditions (**Figures 4E, F**).

### 3.3 Chlorophyll fluorescence and gas exchange

**Figure 5:**
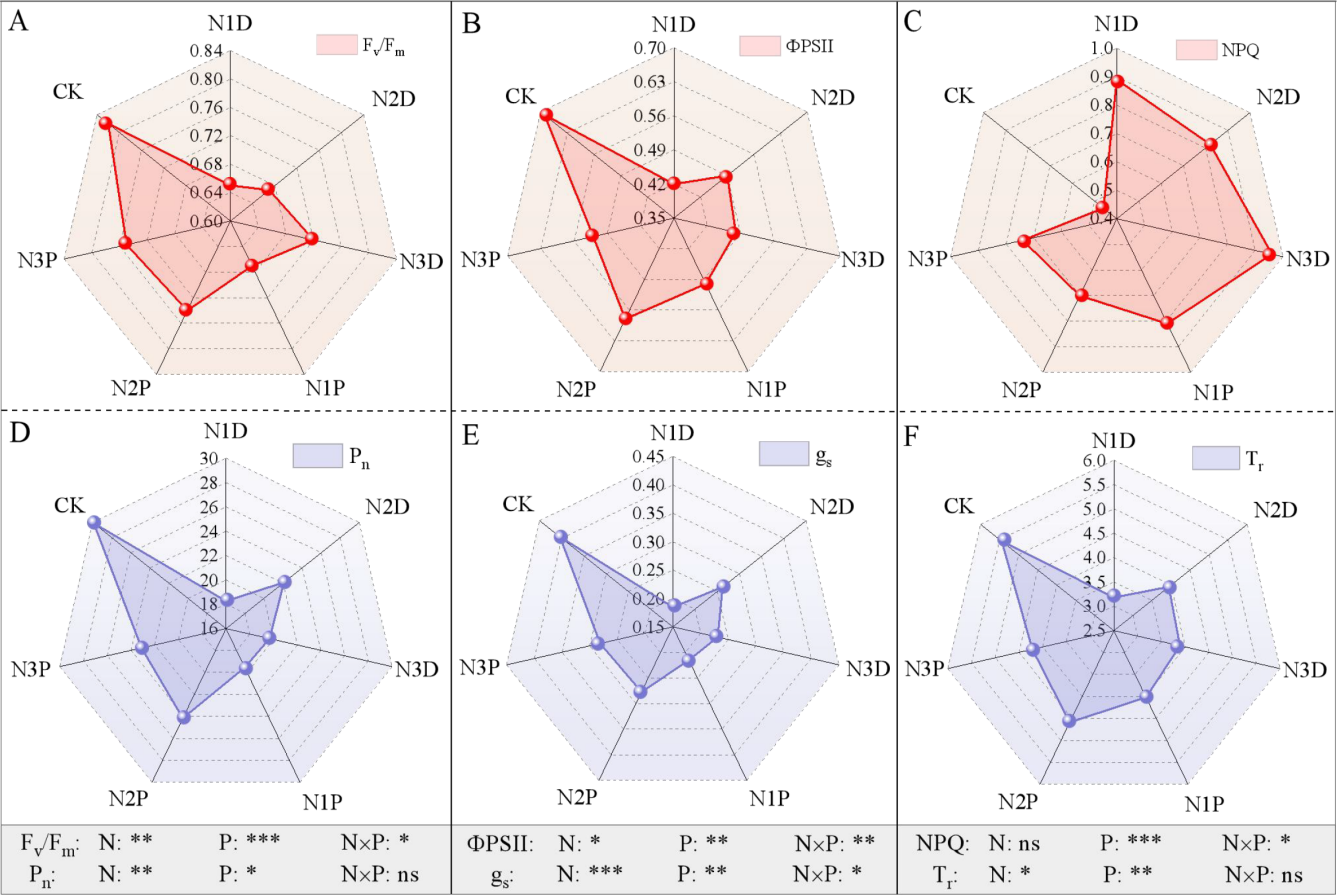
Effects of nitrogen fertilizer and drought priming on the maximum photochemical efficiency of PSII F_v_/F_m_, **(A)**, actual photochemical quantum yield of PSII ΦPSII, **(B)**, non-photochemical quenching coefficient NPQ, **(C)**, net photosynthetic rate P_n_, **(D)**, stomatal conductance g_s_, **(E)**, and transpiration rate T_r_, **(F)** in summer maize. N1D, N1 + no priming + post-anthesis stress; N2D, N2 + no priming + post-anthesis stress; N3D, N3 + no priming + post-anthesis stress; N1P, N1 + drought priming + post-anthesis stress; N2P, N2 + drought priming + post-anthesis stress; N3P, N3 + drought priming + post anthesis stress; CK, N1 + no priming + well-watered; N, nitrogen; P, drought priming. Data represent mean ± SD (F_v_/F_m_, ΦPSII, and NPQ, n = 5; P_n_, g_s_, and T_r_, n=9). *p < 0.05, **p < 0.01, ***p < 0.001, ns is nonsignificant (*p* > 0.05).

The interaction between nitrogen fertilizer (N) and drought priming (P) had a significant impact on maximum photochemical efficiency of PSII (F_v_/F_m_), actual photochemical quantum yield of PSII (ΦPSII), non-photochemical quenching coefficient (NPQ), and stomatal conductance (g_s_) (**Figure 5**). Drought stress led to a marked reduction in F_v_/F_m_ and ΦPSII, with N1D treatment showing 20.7% and 39.1% decreases, respectively, compared with the control. F_v_/F_m_ exhibited an upward trend as N application increased, and this enhancing effect was more pronounced under drought priming. F_v_/F_m_ under N2P and N3P treatments was 13.8% and 15.4% greater than that under N1D treatment, respectively. ΦPSII was markedly higher in the N2P treatment than in the N2D treatment (**Figures 5A, B**). Compared with the N1D, the NPQ under N2P and N3P treatments was reduced by 21.3% and 16.9%, respectively (**Figure 5C**). Notably, the N3 application further exacerbated the reduction in ΦPSII and the increase in NPQ, and ΦPSII in the N3P treatment was 10.3% lower than that in the N1P treatment. Similarly, drought stress induced a significant decrease in gas exchange parameters. The positive regulation of N fertilizer on gas exchange parameters was more pronounced under drought priming conditions, and net photosynthetic rate (P_n_), g_s_, and transpiration rate (T_r_) in the N2P treatment reached 1.32, 1.50, and 1.44 times those in the N1D treatment, respectively (**Figures 5D–F**).

### 3.4 O_2_^-^ and H_2_O_2_ contents and MII

**Figure 6:**
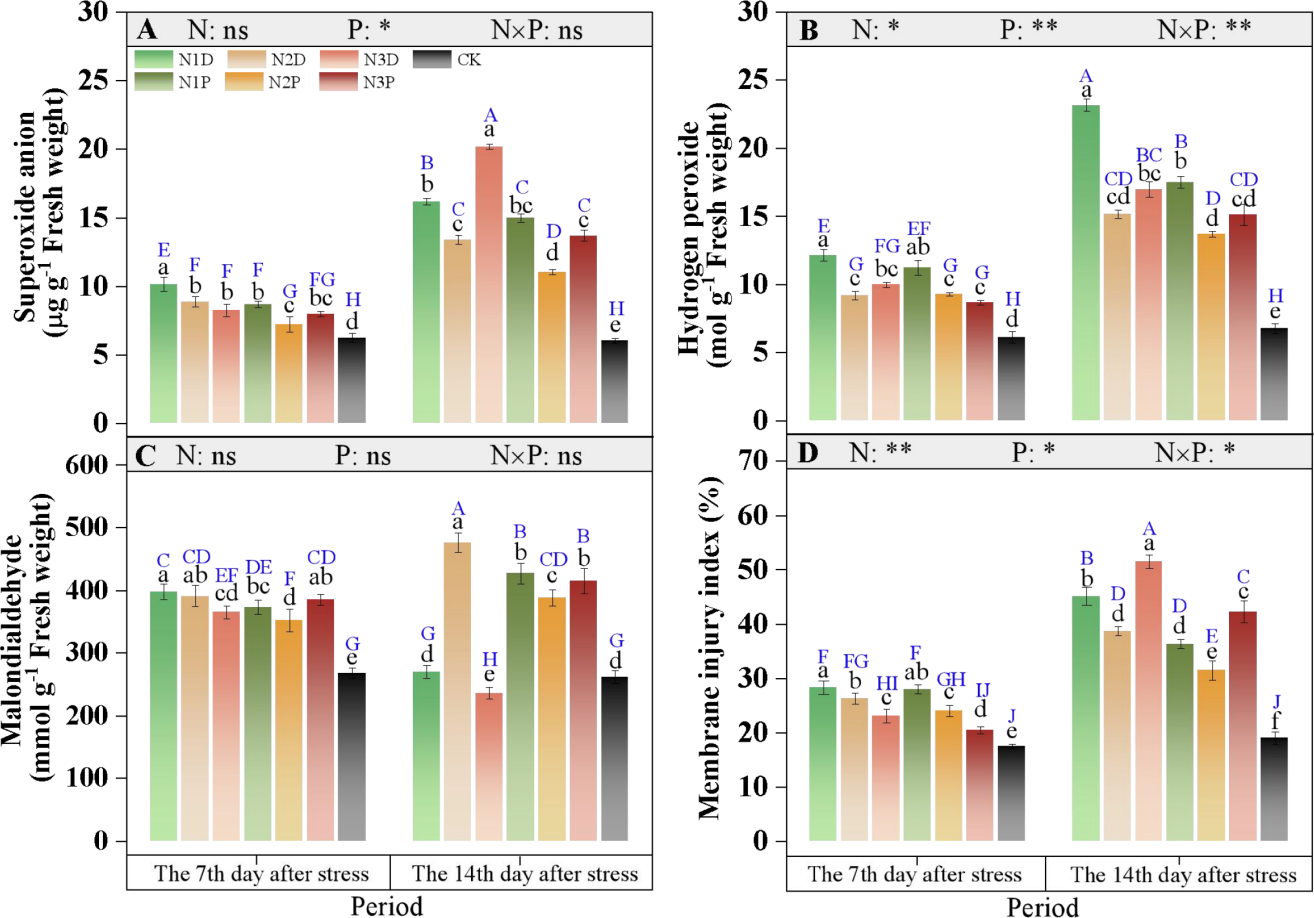
Effects of nitrogen fertilizer and drought priming on superoxide anion O_2_^-^, **(A)** hydrogen peroxide H_2_O_2_, **(B)** malondialdehyde MDA, **(C)** and membrane stability index MII, **(D)** in summer maize. N1D, N1 + no priming + post-anthesis stress; N2D, N2 + no priming + post-anthesis stress; N3D, N3 + no priming + post-anthesis stress; N1P, N1 + drought priming + post-anthesis stress; N2P, N2 + drought priming + post-anthesis stress; N3P, N3 + drought priming + post anthesis stress; CK, N1 + no priming + well-watered; N, nitrogen; P, drought priming. Data represent mean ± SD (n=5). *p < 0.05, **p < 0.01, ***p < 0.001, ns is nonsignificant (*p* > 0.05). Different lowercase letters indicate significant differences (*p* < 0.05) among treatments at the same time point. Different uppercase blue letters indicate significant differences (*p* < 0.05) between the two time points (D7 and D14) across all treatments.

Variations in hydrogen peroxide (H_2_O_2_) content and membrane injury index (MII) were mainly dependent on the interaction between nitrogen fertilizer (N) and drought priming (P) (**Figure 6**). Under no priming conditions, the superoxide anion (O_2_^-^) content decreased progressively as N application increased at D7. At D14, the O_2_^-^ content under N2D treatment was significantly lower than that under N1D and N3D treatments, but was still higher than that under N2P treatment (**Figure 6A**). The H_2_O_2_ content in the N1D treatment surged to 3.4 times that of the control, whereas those in the N2P and N3P treatments were only 2.02- and 2.26-fold higher than that of the control, respectively (**Figure 6B**). At the initial stage of stress, the MDA content was markedly lower in the N3D and N2P treatments than in the N1D. At D14, the MDA content in the N1D and N3D treatments decreased sharply to below the control level, whereas MDA content decreased initially and then increased with increased N supply under priming conditions (**Figure 6C**). For MII, the N3D and N3P treatments showed reductions of 18.4% and 27.6% at D7, respectively, compared with the N1D. At D14, the N2P treatment reduced MII by 30.2% compared with N1D (**Figure 6D**).

### 3.5 The activities of SOD, POD, CAT, and APX

**Figure 7:**
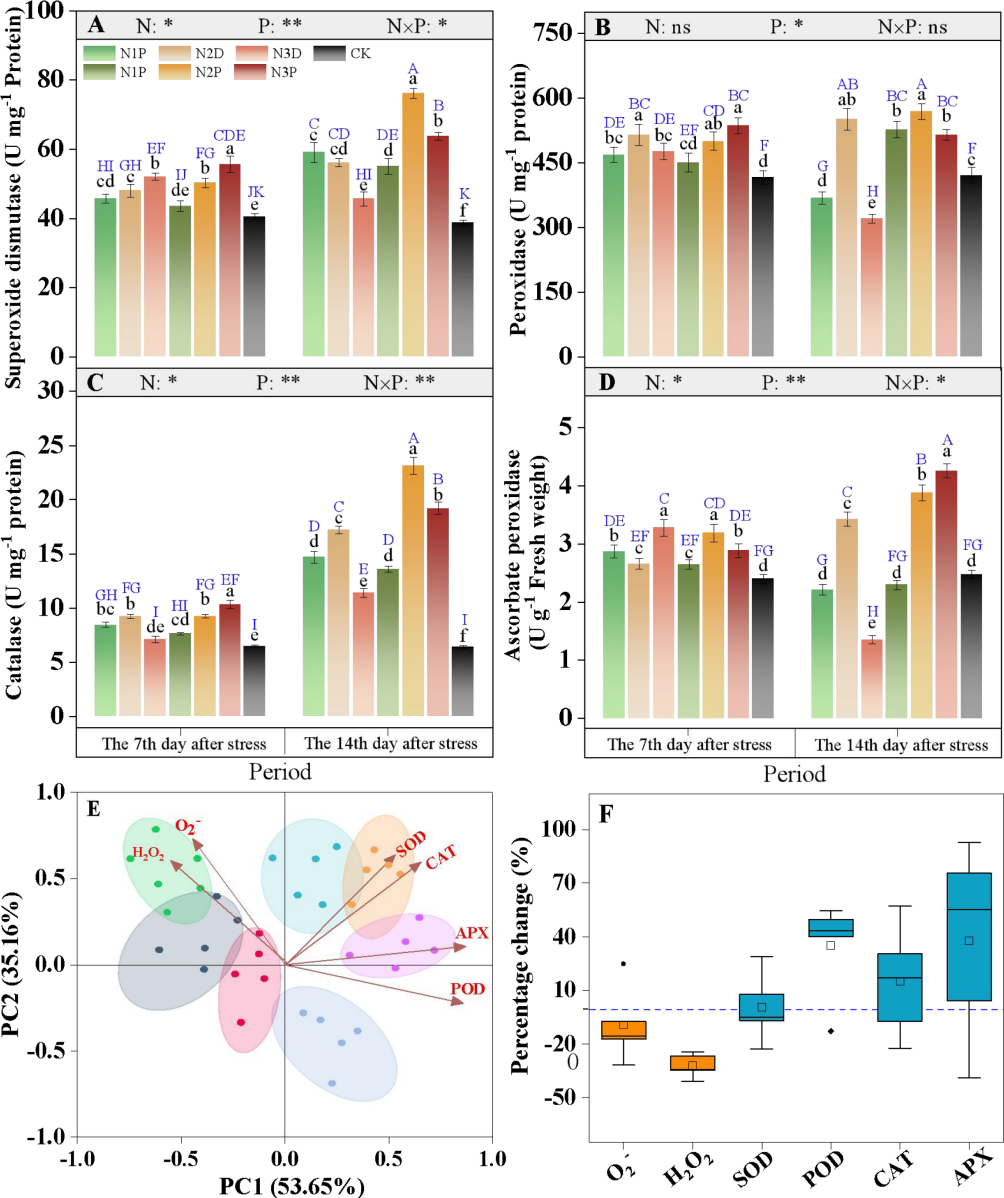
Effects of nitrogen fertilizer and drought priming on superoxide dismutase SOD, **(A)** peroxidase POD, **(B)** catalase CAT, **(C)** and ascorbate peroxidase (APX, **(D)**) activities in summer maize. Principal component analysis (PCA) of reactive oxygen species and antioxidant enzymes **(E)**; Percentage change of reactive oxygen species content and antioxidant enzyme activities **(F)**. The data represent the percentage change of each index relative to the N1D treatment for all treatments (except CK) at D14. N1D, N1 + no priming + post-anthesis stress; N2D, N2 + no priming + post-anthesis stress; N3D, N3 + no priming + post-anthesis stress; N1P, N1 + drought priming + post-anthesis stress; N2P, N2 + drought priming + post-anthesis stress; N3P, N3 + drought priming + post anthesis stress; CK, N1 + no priming + well-watered; N, nitrogen; P, drought priming. Data represent mean ± SD (n=5). *p < 0.05, **p < 0.01, ***p < 0.001, ns is nonsignificant (*p* > 0.05). Different lowercase letters indicate significant differences (*p* < 0.05) among treatments at the same time point. Different uppercase blue letters indicate significant differences (*p* < 0.05) between the two time points (D7 and D14) across all treatments.

The activities of superoxide dismutase (SOD), catalase (CAT), and ascorbate peroxidase (APX) were markedly modulated by the interaction between nitrogen fertilizer (N) and drought priming (P) (**Figure 7**). The antioxidant defense system was notably activated in response to drought stress. At D14, the SOD activity in non-primed plants declined progressively with the elevation of N supply, whereas the N2P and N3P treatments exhibited 28.9% and 7.8% higher SOD activity than the N1D treatment, respectively (**Figure 7A**). At D14, peroxidase (POD) activity in the N1P and N2P treatments was 1.43- and 1.55-fold higher than that in the N1D treatment, respectively. Excessive N application suppressed POD activity, with a more pronounced effect observed in non-primed plants (**Figure 7B**). For CAT activity, the primed plants showed an increase with N application at D7. As stress duration increased, the N2P and N3P treatments exhibited 57.1% and 30.6% higher CAT activity than that of the N1D treatment, respectively (**Figure 7C**). With the prolongation of stress duration, the regulatory effect of N supply and drought priming on ascorbate peroxidase (APX) activity became increasingly evident, with the activities of the N2P and N3P treatments being 1.76 and 1.93 times that of the N1D, respectively (**Figure 7D**).

Principal component analysis revealed that the vectors of antioxidant enzymes formed acute angles, indicating strong positive correlations among these indicators. In contrast, the vectors of H_2_O_2_ and O_2_^-^ were located on the opposite coordinate axis from the antioxidant enzyme vectors, demonstrating significant negative correlations with antioxidant enzyme activities (**Figure 7E**). Compared with non-primed plants, the combined N and priming treatment increased antioxidant enzyme activities by 4.1% to 92.8%, along with a concomitant 7.5% to 40.9% reduction in O_2_^-^ and H_2_O_2_ content (**Figure 7F**).

### 3.6 Pigment metabolism and osmotic regulation substances

#### 3.6.1 Pigment metabolism

**Figure 8:**
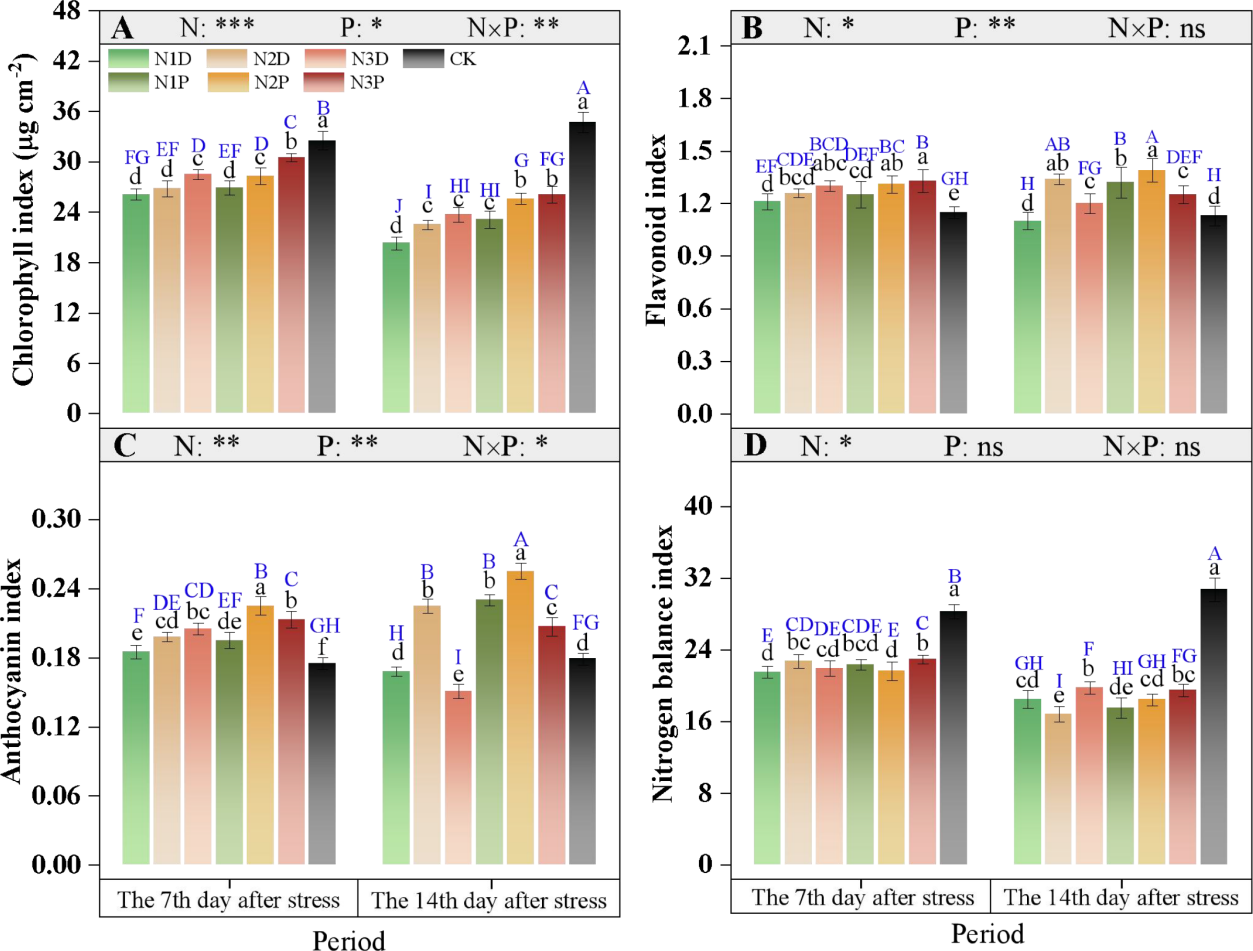
Effects of nitrogen fertilizer and drought priming on chlorophyll index **(A)**, flavonoid index **(B)**, anthocyanin index **(C)**, and N balance index **(D)** in summer maize. N1D, N1 + no priming + post-anthesis stress; N2D, N2 + no priming + post-anthesis stress; N3D, N3 + no priming + post-anthesis stress; N1P, N1 + drought priming + post-anthesis stress; N2P, N2 + drought priming + post-anthesis stress; N3P, N3 + drought priming + post anthesis stress; CK, N1 + no priming + well-watered; N, nitrogen; P, drought priming. Data represent mean ± SD (n=5). *p < 0.05, **p < 0.01, ***p < 0.001, ns is nonsignificant (*p* > 0.05). Different lowercase letters indicate significant differences (*p* < 0.05) among treatments at the same time point. Different uppercase blue letters indicate significant differences (*p* < 0.05) between the two time points (D7 and D14) across all treatments.

The variations in the chlorophyll and anthocyanin indices were mainly affected by the interaction between nitrogen fertilizer (N) and drought priming (P) (**Figure 8**). The leaf chlorophyll index significantly decreased under drought stress conditions, and this inhibitory effect intensified with prolonged stress duration. At D14, the N3D treatment resulted in a markedly higher chlorophyll index than the N1D treatment, whereas that in the N3P treatment was 10.1% higher than that in the N3D treatment (**Figure 8A**). The flavonoid index first increased and then decreased with increasing N application, with the N2P and N2D treatments showing increases of 21.8% and 26.4%, respectively, compared with N1D (**Figure 8B**). For the anthocyanin index, the N3D treatment exhibited a 10.8% increase compared with N1D at D7. At D14, the anthocyanin indices under the N2P, N1P, and N2D treatments were 51.8%, 36.9%, and 33.9% higher than those under the N1D, respectively. Excessive N application inhibited anthocyanin synthesis, and this inhibitory effect was more pronounced without priming (**Figure 8C**). Drought stress exerted a significant inhibitory effect on the N balance index, with a greater reduction observed at D14 than at D7. At D14, N balance index did not differ significantly between the N3D and N3P treatments, but both were higher than those in the other stress treatments (**Figure 8D**).

#### 3.6.2 Osmotic regulation substances

**Figure 9:**
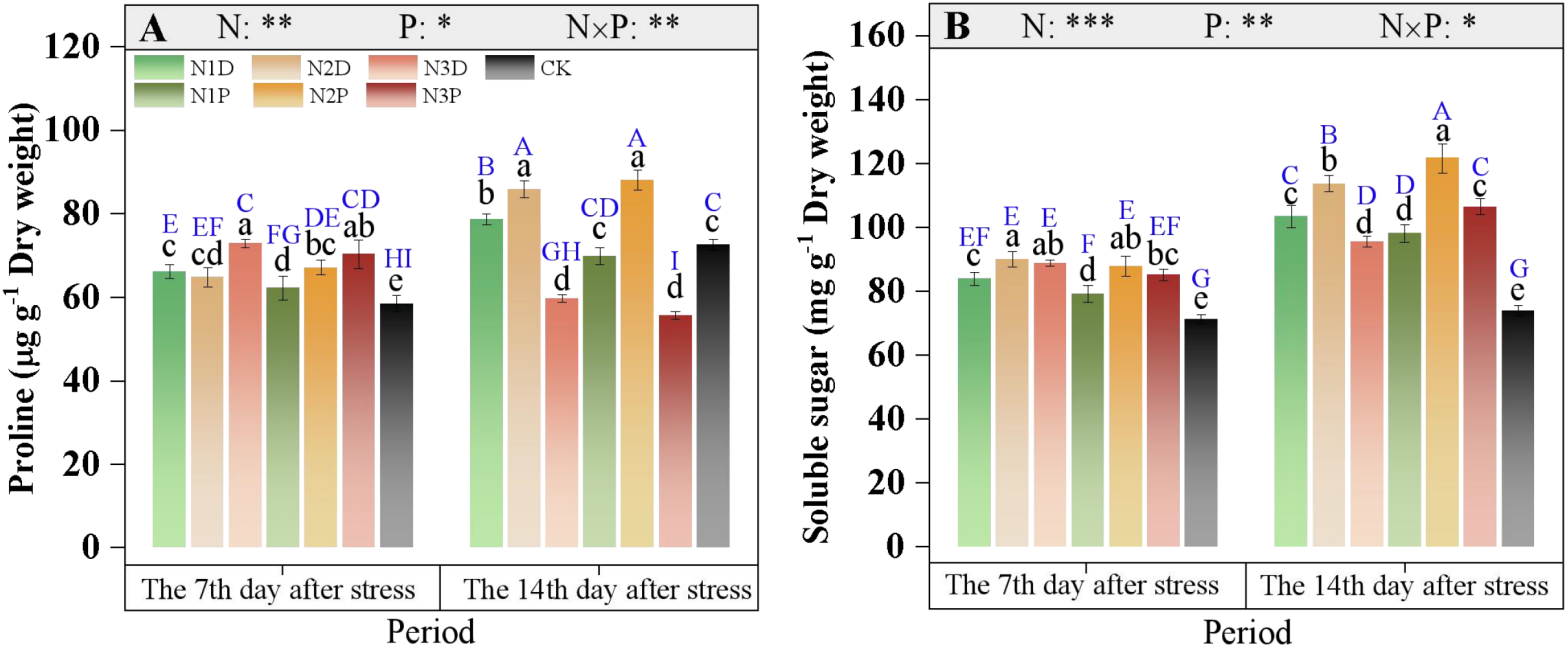
Effects of nitrogen fertilizer and drought priming on the proline **(A)** and soluble sugar **(B)** in summer maize. N1D, N1 + no priming + post-anthesis stress; N2D, N2 + no priming + post-anthesis stress; N3D, N3 + no priming + post-anthesis stress; N1P, N1 + drought priming + post-anthesis stress; N2P, N2 + drought priming + post-anthesis stress; N3P, N3 + drought priming + post anthesis stress; CK, N1 + no priming + well-watered; N, nitrogen; P, drought priming. Data represent mean ± SD (n=5). *p < 0.05, **p < 0.01, ***p < 0.001, ns is nonsignificant (*p* > 0.05). Different lowercase letters indicate significant differences (*p* < 0.05) among treatments at the same time point. Different uppercase blue letters indicate significant differences (*p* < 0.05) between the two time points (D7 and D14) across all treatments.

The proline and soluble sugar contents were affected by the interaction between nitrogen fertilizer (N) and drought priming (P) (**Figure 9**). At D7, the proline and soluble sugar contents increased by 13.0% and 17.7% under drought stress, respectively; under drought priming, proline content increased with increasing nitrogen application rate, while soluble sugar content showed a parabolic trend. At D14, proline content peaked in the N2 treatment, with no significant difference observed between primed and non-primed plants. Excessive N supply led to a reduction in proline levels in the N3P and N3D treatments, whereas the N2P treatment had the highest soluble sugar content, showing a 17.8% increase compared with the N1D (**Figures 9A, B**).

### 3.7 Expression of nitrogen metabolism-related genes

**Figure 10:**
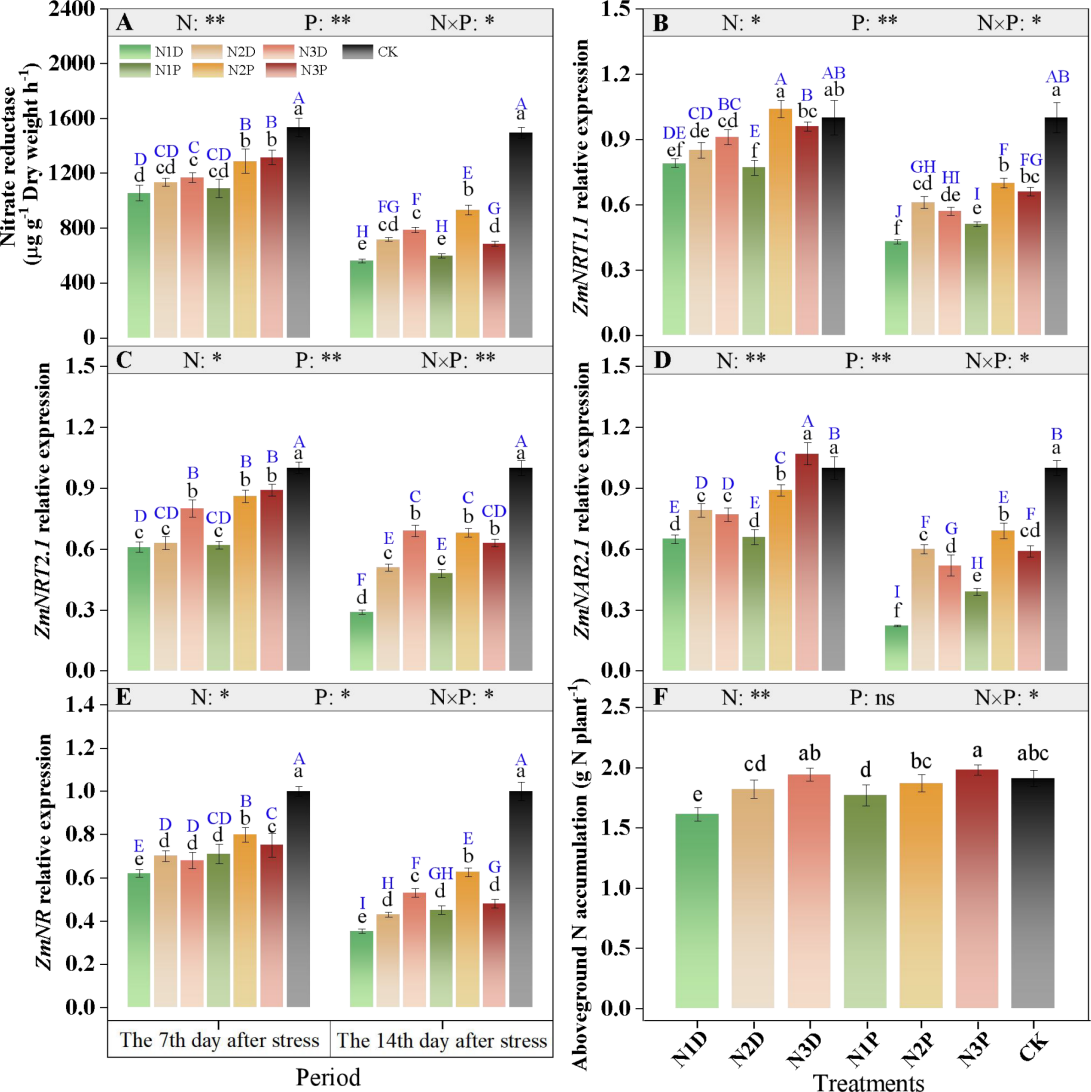
Effects of nitrogen fertilizer and drought priming on nitrate reductase NR, **(A)** activity, ZmNRT1.1 **(B)**, ZmNRT2.1 **(C)**, ZmNAR2.1 **(D)**, ZmNR **(E)**, and aboveground nitrogen accumulation ANA, **(F)** in summer maize. N1D, N1 + no priming + post-anthesis stress; N2D, N2 + no priming + post-anthesis stress; N3D, N3 + no priming + post-anthesis stress; N1P, N1 + drought priming + post-anthesis stress; N2P, N2 + drought priming + post-anthesis stress; N3P, N3 + drought priming + post anthesis stress; CK, N1 + no priming + well-watered; N, nitrogen; P, drought priming. Data represent mean ± SD (n=5). *p < 0.05, **p < 0.01, ***p < 0.001, ns is nonsignificant (*p* > 0.05). Different lowercase letters indicate significant differences (*p* < 0.05) among treatments at the same time point. Different uppercase blue letters indicate significant differences (*p* < 0.05) between the two time points (D7 and D14) across all treatments.

Nitrate reductase (NR) activity and the expression levels of nitrogen metabolism-related genes were significantly affected by the interaction between nitrogen fertilizer (N) and drought priming (P). NR activity in the N1D treatment was 31.1% lower than that of the control, whereas the N2P and N3P treatments exhibited only 16.0% and 14.1% decreases in NR activity compared with the control. At D14, NR activity in the N2P treatment was 1.66 times higher than that in the N1D treatment (**Figure 10A**). Drought stress suppressed the expression of key N metabolism-related genes. At D14, the N2D and N3D treatments maintained relatively high ZmNRT1.1 expression levels, but these levels remained notably lower than those in the N2P treatment (**Figure 10B**). The ZmNRT2.1 expression level in the N1D treatment decreased to 29.4% of that in the control at D14, and significant differences in ZmNRT2.1 expression were observed among the N3D, N2P, and N3P plants (**Figure 10C**).

Under drought priming conditions, increased N application induced ZmNAR2.1 expression at D7, with the expression levels in the N2P and N3P being 36.9% and 64.6% higher than those in the N1D treatment, respectively. At D14, ZmNAR2.1 expression in the N1D treatment decreased to 77.7% of the control, whereas that in the N2P treatment only decreased to 31.0%. At the initial stage of stress, primed plants exhibited higher expression levels than non-primed plants under the same N supply (**Figure 10D**). Prolonged stress led to a continuous decline in ZmNR expression; the ZmNR expression level under the N1D treatment was 35.2% of the control, whereas those under the N3D and N2P were 53.0% and 62.6% of the control, respectively (**Figure 10E**). Aboveground N accumulation (ANA) under N1D treatment was 15.7% lower than that under the control. ANA increased with increasing N application, particularly under priming conditions. The ANA in the N2P and N3P treatments increased by 16.1% and 23.0%, respectively,compared with the N1D (**Figure 10F**).

### 3.8 Drought tolerance

**Figure 11:**
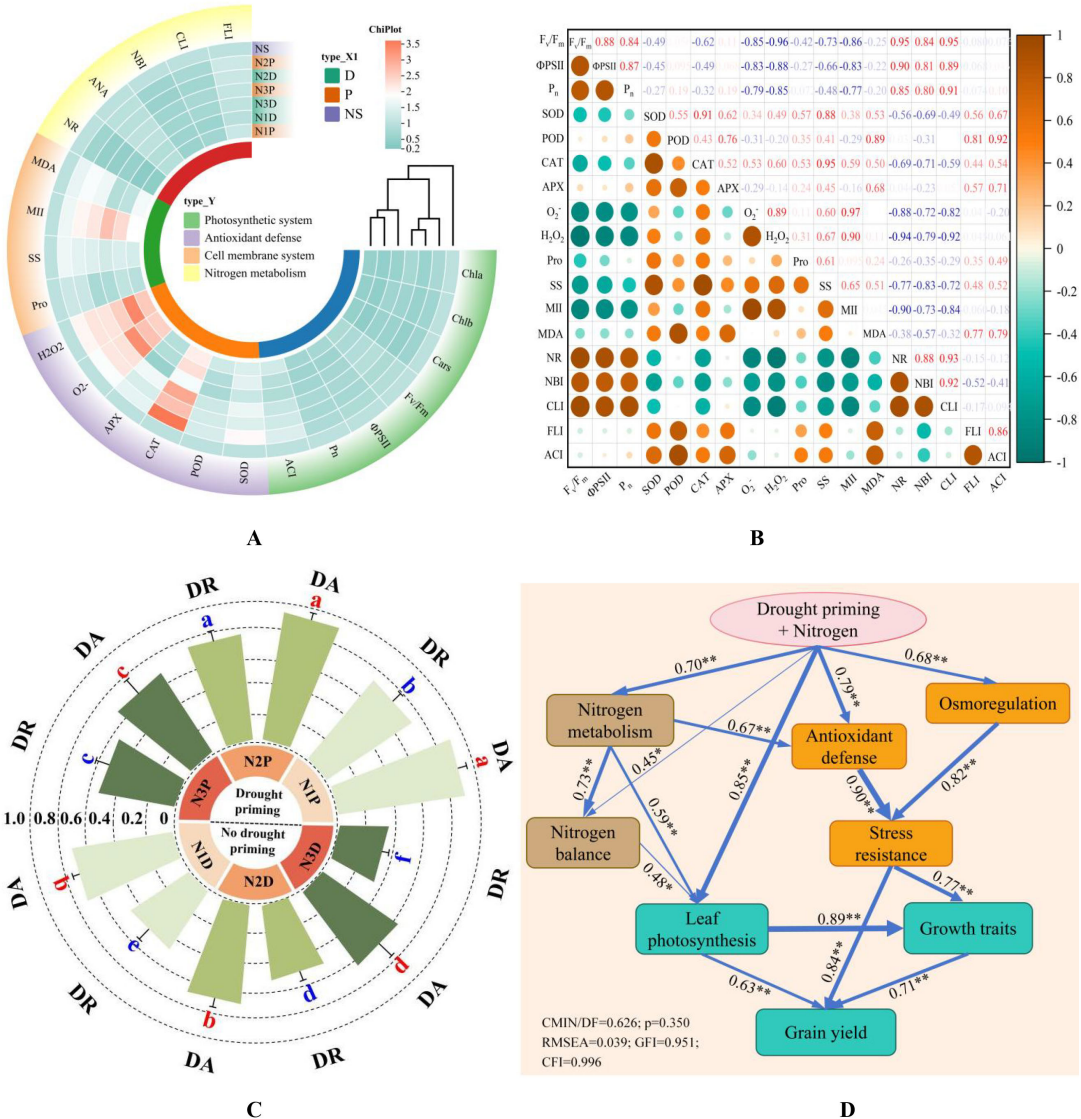
Heatmap analysis of physiological indices under N fertilizer and drought priming regulation **(A)**; Correlation analysis of physiological indices **(B)**; Evaluation of drought resistance (DR) and drought adaptability (DA) in maize plants **(C)**; Structural equation modeling assessing the direct and indirect effects of drought priming combined with N fertilizer **(D)**. Pro, proline; SS, soluble sugar; CLI, chlorophyll index; ACI, anthocyanin index; FLI, flavonoid index; NBI, N balance index; MII, membrane injury index. Numbers adjacent to arrows represent standardized path coefficients, indicating the change in the dependent variable (in standard deviations) per one standard deviation change in each independent variable The arrowed lines denote the hypothesized causal relationship between the origin of the arrow (the cause) and the endpoint of the arrow (the effect).

Heatmap analysis revealed that compared with N1D treatment, the gas exchange parameters, chlorophyll fluorescence parameters, chlorophyll index, and ANA in the N2P and N3P treatments were maintained at relatively healthy levels, and the N2P and N3P plants exhibited stronger antioxidant capacity. The N2P plants showed higher FLI, ACI, proline and soluble sugar contents, and NR activity, while maintaining lower levels of O_2_^–^, H_2_O_2_, MDA, and MII (**Figure 11A**). F_v_/F_m_ and ΦPSII were strongly positively correlated with NR, CLI, and P_n_. P_n_ displayed a significant positive correlation with NR, NBI, and CLI but was negatively correlated with O_2_^–^ and H_2_O_2_. All antioxidant enzyme activities showed clear positive correlations with FLI and ACI. Proline had the strongest correlation with SS, whereas SS exhibited significant negative correlations with NR, NBI, and CLI. NR activity exhibited a strong positive correlation with NBI and CLI (**Figure 11B**).

The N2P treatment exhibited the highest drought resistance (0.721), followed by N1P (0.672). Drought adaptability did not differ significantly between these two treatments, but both were significantly superior to the other treatments (**Figure 11C**). The synergistic regulation of N and drought priming had substantial positive effects on N metabolism, photosynthesis, antioxidant activity, and osmotic adjustment capacity. Improved N metabolism enhances photosynthetic capacity, further promoting yield increase by positively regulating plant growth and development. Antioxidant defense and osmotic adjustment had significant positive effects on stress tolerance, and the enhancement of stress tolerance exerted direct or indirect positive effects on maize yield (**Figure 11D**).

## 4 Discussion

### 4.1 Effects of drought priming and N fertilizer on maize growth and yield formation of maize

Aboveground growth and root morphology are key phenotypes of summer maize in response to drought stress after anthesis. Their synergistic state directly affects the plant’s resource acquisition capacity and photosynthetic product supply efficiency, thereby determining yield levels (Dong et al., 2022; Yue et al., 2022). Under post-anthesis drought stress, drought stress significantly reduced plant height, leaf area, and aboveground dry matter accumulation in maize by inhibiting the synthesis of auxin and gibberellin while promoting abscisic acid accumulation (Lamlom et al., 2025). Drought stress also inhibited root cell division and elongation, leading to significant reductions in root length density and root surface area density (Li et al., 2025). The dual inhibition of aboveground and root growth ultimately resulted in significant decreases in the number of kernels per ear, 1000-kernel weight, and grain yield. This is mainly because drought disrupts the structural integrity of the photosynthetic system, causing a substantial decline in the photosynthetic rate and an insufficient carbohydrate supply required for grain filling (Pokhrel, 2021). Additionally, drought stress downregulated the expression of key genes involved in N metabolism and reduced NR activity, thereby hindering N translocation to the ears and further affecting grain filling.

Although N application can mitigate the yield reduction caused by drought stress by optimizing yield components, its regulatory effect was influenced by drought priming. This study found that the combined treatment of priming + N fertilizer demonstrated significantly superior effects compared to individual treatments, with most yield-related traits peaking at moderate N application. This finding aligns with the conclusions of Ullah et al. (2022), indicating that wheat plants subjected to drought priming combined with an appropriate N supply exhibit greater sink capacity and higher photosynthate translocation efficiency, which are key mechanisms that ensure normal grain development and reduce yield losses. Based on the SEM model analysis constructed in this study (**Figure 11D**), drought priming combined with N supply can enhance plant drought resistance by promoting N metabolism, further optimizing N balance, and strengthening antioxidant defense and osmotic adjustment capacity, thereby maintaining growth traits and ultimately improving yield. Moreover, combined treatment significantly improved photosynthesis, providing sufficient carbon assimilation products for yield formation (Chen et al., 2025). These regulatory pathways exhibited the strongest synergistic effects under N2 supply.

From a physiological perspective, drought priming combined with N2 optimized root architecture, thereby enhancing water and nutrient uptake capacity and providing a resource foundation for maintaining aboveground physiological functions (Liu et al., 2023). This treatment promoted chlorophyll synthesis and sustained higher photosynthetic efficiency (**Figure 11D**), supplying adequate photosynthetic products for root growth and achieving coordinated recovery between belowground and aboveground parts. These results were consistent with those of Maywald et al. (2022), who reported that N supplementation combined with irrigation during stress recovery significantly improved maize growth performance under repeated drought conditions. The optimized growth status achieved by this treatment provided a material foundation for grain filling, while enhanced N metabolism improved the translocation efficiency of assimilates to grains, collectively driving yield improvement. It is noteworthy that excessive N application exacerbated water stress due to excessive vegetative growth, disrupting the carbon-N metabolic balance (Kong et al., 2017). Although it slightly increased the harvest index, it failed to achieve positive synergism among various physiological processes in the model (**Figure 11D**), making it difficult to simultaneously optimize growth and yield. In summary, drought priming combined with N2 application constructed a physiological regulatory network that efficiently promoted yield enhancement by synergistically optimizing multiple pathways, including N metabolism, photosynthesis, and stress resistance physiology.

### 4.2 Effects of drought priming and N fertilizer on chlorophyll fluorescence, photosynthesis and pigment metabolism

Post-anthesis drought stress inhibits the photosynthetic system via stomatal and non-stomatal limitations. Drought stress reduces stomatal conductance and intercellular CO_2_ concentration, leading to a significant decline in the net photosynthetic rate (Chen et al., 2023; Zahra et al., 2023). In terms of non-stomatal limitations, drought induces massive ROS accumulation, which damages chloroplast structure and inactivates photosynthetic enzymes (Hu et al., 2023), triggering photoinhibition of photosystem II. This was manifested as reduced F_v_/F_m_ and ΦPSII, with excess light energy dissipated as heat, resulting in a significant decrease in the photochemical energy available for carbon assimilation (Javornik et al., 2023). Concurrently, excessive ROS accumulation hinders chlorophyll synthesis and accelerates its degradation, causing a decline in chlorophyll indices and further weakening the light-harvesting capacity. To cope with drought stress, plants activate secondary metabolic compensation by increasing the flavonoid and anthocyanin content, thereby enhancing the ROS-scavenging capacity and absorbing excess light energy, thereby achieving dual defense of photoprotection and antioxidation (Kurowska et al., 2025).

Under drought priming, moderate N application alleviated the aforementioned stomatal and non-stomatal limitations. This treatment mitigated stomatal closure by optimizing root water uptake, thereby reducing stomatal limitation. Based on stress memory, N promotes the precise allocation of N to the photosynthetic system, optimizes the expression of proteins related to photosynthetic state transition, and reduces excess excitation energy in PSII (Du et al., 2020; Tankari et al., 2021; Kaya and Adamakis, 2025), thereby alleviating photoinhibition damage (Abid et al., 2016). Additionally, N2 supply combined with drought priming maintained a higher chlorophyll index by scavenging ROS and directing N allocation to chloroplast repair and chlorophyll synthesis. Stress memory induced by priming upregulated the expression of key enzyme genes in phenylpropanoid metabolism, and N supply provided the N skeleton for phenolic synthesis (Yin et al., 2025; Zhi et al., 2025), promoting the accumulation of flavonoids and anthocyanins and further enhancing photoprotection and antioxidant functions. These results were consistent with the conclusions of Ullah et al. (2022), who reported that drought priming mitigated the adverse effects of subsequent drought on photosynthesis by activating stress memory. The present study found that under priming conditions, N3 application significantly exacerbated the reduction in ΦPSII and the increase in NPQ. This was mainly attributed to the fact that excessive N application consumed large amounts of reducing power and ATP for N assimilation, thereby disrupting the energy metabolism balance in chloroplasts (Shi et al., 2020). This was consistent with the findings of Song et al. (2018), who suggested that ΦPSII initially increased and then decreased under sustained drought stress, indicating that excessive N concentration inhibited light absorption and conversion efficiency in maize.

### 4.3 Effects of drought priming and N fertilizer on membrane damage and antioxidant enzyme activities

Under post-anthesis drought stress, impaired function of the photosynthetic system and electron transport chain triggers excessive ROS accumulation, thereby disrupting cellular redox homeostasis and causing severe damage to membrane structures (Ru et al., 2023; Gelaw and Sanan-Mishra, 2024). The antioxidant enzyme system serves as a core defense mechanism for plants to scavenge ROS and resist membrane lipid peroxidation, and the dynamic balance between this system and ROS levels determines the extent of oxidative damage in plants. This study showed that drought stress significantly increased O_2_^–^ and H_2_O_2_ levels in leaves, accompanied by a sharp rise in the membrane injury index. Although plants enhanced the activities of SOD, POD, CAT, and APX through activating stress responses, the increase in enzyme activities induced by drought alone was limited and insufficient to completely eliminate the over-accumulated ROS, resulting in significant oxidative damage.

The combined application of drought priming and N2 synergistically elevated antioxidant enzyme activities, establishing a more efficient oxidative defense system that enabled continuous ROS scavenging and effective protection of membrane integrity. Compared to the individual treatments, this combination markedly increased the activities of the four antioxidant enzymes, and rapidly decomposed excess O_2_^–^ and H_2_O_2_ into harmless substances, thereby effectively inhibiting membrane lipid peroxidation and maintaining membrane integrity. The underlying mechanisms may be as follows: stress memory induced by drought priming pre-activated the expression of genes related to antioxidant enzymes, enabling plants to rapidly initiate defense systems upon subsequent drought exposure (Peer et al., 2025). Meanwhile, sufficient N supply further amplifies the stress memory effect by upregulating the expression of N metabolism-related genes, enhancing N uptake and utilization, and promoting the reconstruction of cellular redox homeostasis (Gelaw et al., 2023). Ullah et al. (2022) proposed that N fertilizer could improve the drought tolerance of primed plants, which further supports the results of the present study. Although moderate N application helped maintain a more stable antioxidant system, this study found that excessive N application inhibited this effect. Excessive N supply leads to excessive vegetative growth, increases water consumption, and subsequently exacerbates plant water stress, resulting in a significant increase in H_2_O_2_ and O_2_^–^ content. Similarly, Zhang et al. (2022) reported that excessive nitrogen application aggravates membrane lipid peroxidation in tomato plants, significantly reduces SOD and POD activity, and weakens the function of the antioxidant system.

### 4.4 Effects of drought priming and N fertilizer on osmoregulatory substances and N metabolism gene expression

Osmoregulation is a crucial physiological adaptation strategy for crops to resist drought stress (Abdallah et al., 2017; Ru et al., 2022), while N metabolism serves as a core pathway for plant growth, development, and stress response, providing key metabolic substrates for osmoregulation through its molecular regulation. This study observed that under post-anthesis drought stress, the proline and soluble sugar contents in maize leaves significantly increased. Proline accumulated rapidly through the activation of biosynthetic pathways and inhibition of degradation pathways, while soluble sugars functioned in both osmoregulation and carbon storage. Their synergistic action reduces cellular osmotic potential, helping maintain cellular water balance and stabilizing proteins and membrane structures (Abdallah et al., 2017; Perez et al., 2010). However, drought stress significantly suppressed N metabolism processes, manifested as the downregulation of ZmNRT1.1, ZmNRT2.1, ZmNAR2.1, and ZmNR expression, as well as reduced NR activity, leading to substantial declines in N uptake and assimilation efficiency (Dubey et al., 2021). The suppression of N metabolism further restricted the supply of N substrates required for multiple stress-resistant physiological processes, limiting the synthesis of osmoregulatory substances, and thus weakening the overall stress tolerance of plants.

The combination of drought priming and N2 activated the synthesis pathways of osmoregulatory substances through stress memory, enabling plants to respond rapidly when stress reoccurred. Meanwhile, sufficient N supply provided essential carbon skeletons and N substrates for proline and soluble sugar synthesis, significantly promoting their accumulation (Wang et al., 2018) and enhancing cellular water retention capacity. Concurrently, this combined treatment effectively reversed the suppression of key N metabolism gene expression, thereby improving NR activity and N use efficiency in grains. We speculate that drought priming may pre-trigger stress memory by activating ABA-related signaling pathways, enhancing the plant’s N uptake potential (Zhang et al., 2019), while optimal N supply further improves plant N nutritional status and supports the synthesis of signaling molecules and consolidation of stress memory. Therefore, enhanced N metabolism not only directly guarantees the raw materials for osmoregulatory substance synthesis, but also forms a positive regulatory cycle for the osmoregulatory system by maintaining cellular metabolic homeostasis and signal transduction efficiency (Ahmad et al., 2023).

### 4.5 Comprehensive evaluation of drought tolerance and regulatory mechanisms

This study confirmed that the management strategy combining drought priming with appropriate N supply enhanced post-anthesis drought tolerance in summer maize, and its core physiological basis lies in the activation of plant N metabolism. This strategy upregulated the expression of ZmNRT1.1, ZmNAR2.1, and ZmNR, significantly improving N uptake and assimilation efficiency. Enhanced N metabolism serves as a regulatory hub that drives the synergistic optimization of multiple physiological processes. First, the directional allocation of N to the photosynthetic system ensures chlorophyll synthesis and stability of the photosynthetic apparatus, maintaining a high P_n_. This provides a stable supply of assimilates for stress resistance metabolism and yield formation (Yue et al., 2021; Mu and Chen, 2021). Second, the allocation of N to the antioxidant system provides substrates for the synthesis of key antioxidant enzymes, effectively enhancing their activities, enabling timely scavenging of drought-induced ROS, and protecting the integrity of cell membranes and photosynthetic membranes.

More importantly, these physiological processes form a synergistic network characterized by mutual support and positive feedback (**Figure 11D**). The antioxidant system effectively protects the activities of enzymes related to photosynthesis and N metabolism. Cellular water homeostasis maintained by osmoregulation provides a suitable environment for physiological processes, and efficient photosynthesis in turn supplies carbon skeletons for N metabolism and the synthesis of stress-resistant substances. Ultimately, under the regulation of N metabolism, this multi-level synergistic mechanism optimize the water and N use efficiency and maintained the dynamic balance of carbon-N metabolism as well as cellular homeostasis under post-anthesis drought stress, thereby significantly enhancing drought tolerance and reducing yield loss (Ren et al., 2020).

In regions prone to post-anthesis drought, adopting the agronomic management strategy of drought priming combined with appropriate N fertilizer can simultaneously activate the plant’s drought stress memory and N metabolism regulatory network. Through the synergistic optimization of multiple physiological processes, improvements in drought tolerance, yield, and water-N use efficiency were achieved. Furthermore, this study identified the optimal threshold for N fertilizer regulation, providing important theoretical foundations and technical support for the stable and high-efficiency cultivation of summer maize in arid and semi-arid regions. However, it must be acknowledged that the present findings are primarily based on a single-season pot experiment in a rain-shelter. While this controlled setup was essential for accurately isolating soil moisture and nitrogen levels without confounding external factors like natural rainfall and microenvironmental differences, actual agricultural ecosystems are highly dynamic. Climatic variations across years and field heterogeneity may alter the interaction between drought priming and nitrogen supply. Consequently, it is necessary for future studies to conduct multi-year field trials to verify the inter-seasonal stability and practical viability of this synergistic water-nitrogen strategy, which will provide a solid theoretical basis for its broad application in summer maize production in arid and semi-arid areas.

## 5 Conclusion

This study demonstrated that drought priming alone only partially mitigated the damage induced by post-anthesis drought stress in summer maize, whereas the combined application of drought priming with moderate nitrogen supply significantly enhanced the drought-resistant effect. Specifically, this combination effectively mitigated the inhibitory effect of drought on the maximum photochemical efficiency of PSII and actual photochemical quantum yield of PSII, and markedly increased the chlorophyll, flavonoid, and anthocyanin indices, thereby maintaining a higher net photosynthetic rate under stress. Meanwhile, nitrogen application strengthened the activation of the antioxidant system by drought priming, effectively reducing hydrogen peroxide and superoxide anion accumulation, and improving membrane injury index to alleviate oxidative damage. Furthermore, the regulation of priming and nitrogen fertilizer maintained high expression levels of ZmNRT1.1, ZmNRT2.1, ZmNAR2.1, and ZmNR, enhanced nitrate reductase activity and aboveground nitrogen accumulation, and optimized root density-related traits to improve water and nitrogen uptake and utilization efficiency. The synergistic improvement in growth and physiological functions collectively enhanced the drought resistance of summer maize, culminating in a 16.2% higher grain yield in the N2P treatment than in the N1D treatment under post-anthesis drought stress. Future research could be extended to different maize genotypes and drought stress gradients to clarify the universality of this water-nitrogen regulation strategy.

## Data Availability

The original contributions presented in the study are included in the article/**Supplementary Material**. Further inquiries can be directed to the corresponding author.
