# Supplementary material for: Nitrogen fertilizer modulated the effect of drought priming on photosynthesis, antioxidant defense, nitrogen metabolism, yield in summer maize
Source: Front Plant Sci. 2026 Apr 13;17:1792261. doi: 10.3389/fpls.2026.1792261 (PMC13111099; doi:10.3389/fpls.2026.1792261)
Supplement: Supplementary file 1 [file DataSheet1.pdf]

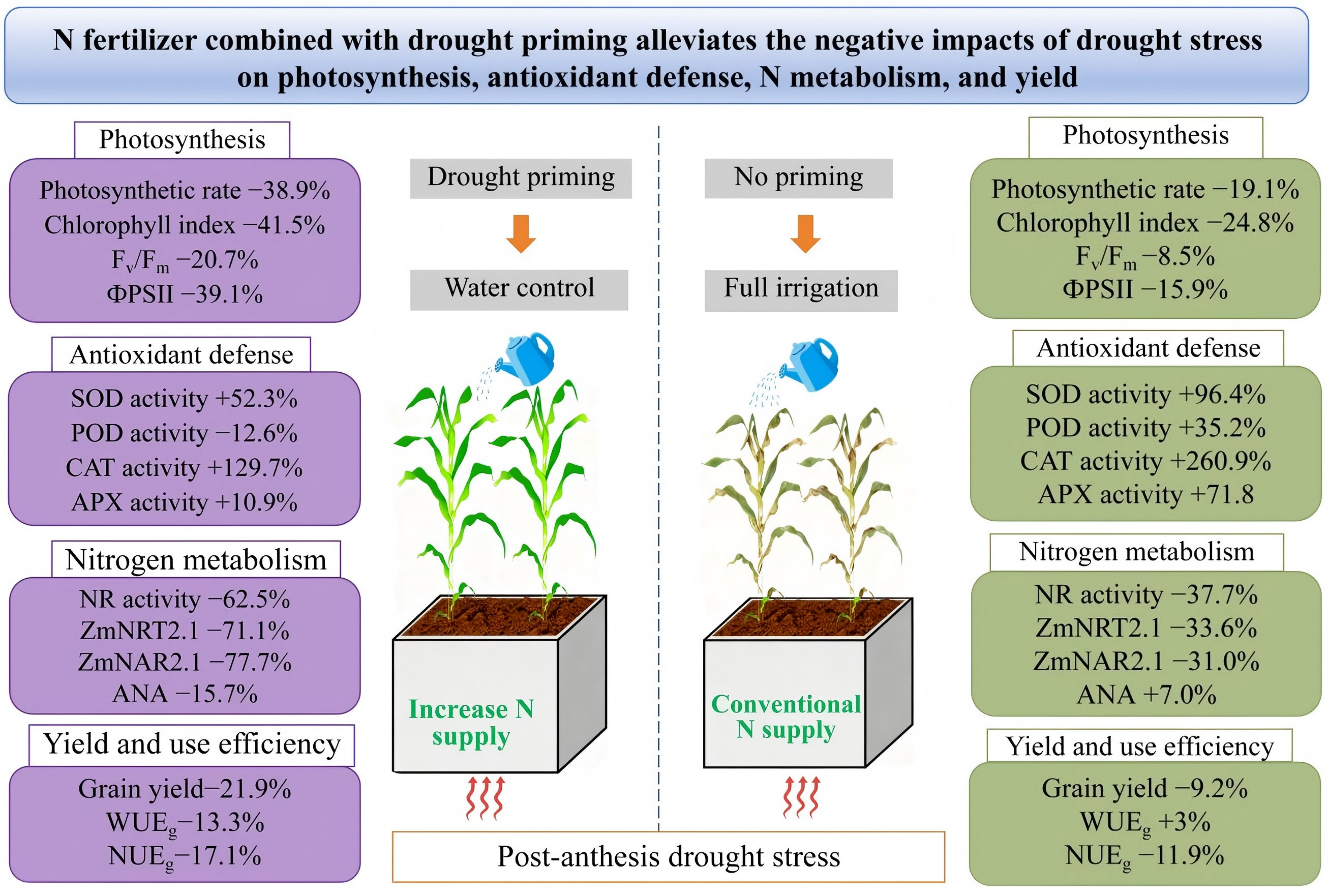
N fertilizer combined with drought priming alleviates the negative impacts of drought stress on photosynthesis, antioxidant defense, N metabolism, and yield
Photosynthesis
Photosynthetic rate −38.9%
Chlorophyll index −41.5%
F_v/F_m −20.7%
ΦPSII −39.1%
Antioxidant defense
SOD activity +52.3%
POD activity −12.6%
CAT activity +129.7%
APX activity +10.9%
Nitrogen metabolism
NR activity −62.5%
ZmNRT2.1 −71.1%
ZmNAR2.1 −77.7%
ANA −15.7%
Yield and use efficiency
Grain yield−21.9%
WUE_g−13.3%
NUE_g−17.1%
Drought priming
Water control
Increase N supply
No priming
Full irrigation
Conventional N supply
Post-anthesis drought stress
Photosynthesis
Photosynthetic rate −19.1%
Chlorophyll index −24.8%
F_v/F_m −8.5%
ΦPSII −15.9%
Antioxidant defense
SOD activity +96.4%
POD activity +35.2%
CAT activity +260.9%
APX activity +71.8
Nitrogen metabolism
NR activity −37.7%
ZmNRT2.1 −33.6%
ZmNAR2.1 −31.0%
ANA +7.0%
Yield and use efficiency
Grain yield −9.2%
WUE_g +3%
NUE_g −11.9%
