# Supplementary material for: Nitrogen fertilizer modulated the effect of drought priming on photosynthesis, antioxidant defense, nitrogen metabolism, yield in summer maize
Source: Front Plant Sci. 2026 Apr 13;17:1792261. doi: 10.3389/fpls.2026.1792261 (PMC13111099; doi:10.3389/fpls.2026.1792261)
Supplement: Supplementary file 2 [file Table1.docx]

Supplementary Table 1. Primers used in this study

| Primer Name | Primer sequence- 5’ to 3’ | Tm (℃) | Product length (bp) |
| --- | --- | --- | --- |
| ZmNRT2.1-F | ATCTTCGGGGTCCATCCCCTTGT | 58 | 120 |
| ZmNRT2.1-R | CAGCGTGCACGCCATGATCAT | 58 | 120 |
| ZmNRT1.1/NPF6.3-F | CCGCCTATGAAATCGTCCTAAT | 58 | 115 |
| ZmNRT1.1/NPF6.3-R | GACCGTGTTCGAGGTACGACCC | 58 | 115 |
| ZmNAR2.1-F | CTCGCCTTCTTCTTCGTCATT | 58 | 130 |
| ZmNAR2.1-R | ATCAGCAACGACAGCCACTG | 58 | 130 |
| ZmNR-F | GGTGAAGATCAACGCGTGCAA | 58 | 125 |
| ZmNR-R | ATGTCTCGAGGTGCTTCTG | 58 | 125 |
